# Ablation Modalities for Therapeutic Intervention in Arrhythmia-Related Cardiovascular Disease: Focus on Electroporation

**DOI:** 10.3390/jcm10122657

**Published:** 2021-06-16

**Authors:** Shauna McBride, Sahar Avazzadeh, Antony M. Wheatley, Barry O’Brien, Ken Coffey, Adnan Elahi, Martin O’Halloran, Leo R. Quinlan

**Affiliations:** 1 Physiology and Cellular Physiology Laboratory, CÚRAM SFI Centre for Research in Medical Devices, School of Medicine, Human Biology Building, National University of Ireland (NUI) Galway, H91 W5P7 Galway, Ireland; shauna.mcbride@nuigalway.ie (S.M.); sahar.avazzadeh@nuigalway.ie (S.A.); antony.wheatley@nuigalway.ie (A.M.W.); 2 AtriAN Medical Limited, Unit 204, NUIG Business Innovation Centre, Upper Newcastle, H91 R6W6 Galway, Ireland; barry.obrien@atrianmedical.com (B.O.); ken.coffey@atrianmedical.com (K.C.); 3 Translational Medical Device Lab (TMDL), Lamb Translational Research Facility, University College Hospital Galway, H91 V4AY Galway, Ireland; adnan.elahi@nuigalway.ie (A.E.); martin.ohalloran@nuigalway.ie (M.O.); 4 Electrical & Electronic Engineering, School of Engineering, National University of Ireland Galway, H91 HX31 Galway, Ireland; 5 CÚRAM, SFI Research Centre for Medical Devices, National University of Ireland Galway, H92 W2TY Galway, Ireland

**Keywords:** electroporation, pulsed field ablation, cardiac, heart, arrhythmia, atrial fibrillation

## Abstract

Targeted cellular ablation is being increasingly used in the treatment of arrhythmias and structural heart disease. Catheter-based ablation for atrial fibrillation (AF) is considered a safe and effective approach for patients who are medication refractory. Electroporation (EPo) employs electrical energy to disrupt cell membranes which has a minimally thermal effect. The nanopores that arise from EPo can be temporary or permanent. Reversible electroporation is transitory in nature and cell viability is maintained, whereas irreversible electroporation causes permanent pore formation, leading to loss of cellular homeostasis and cell death. Several studies report that EPo displays a degree of specificity in terms of the lethal threshold required to induce cell death in different tissues. However, significantly more research is required to scope the profile of EPo thresholds for specific cell types within complex tissues. Irreversible electroporation (IRE) as an ablative approach appears to overcome the significant negative effects associated with thermal based techniques, particularly collateral damage to surrounding structures. With further fine-tuning of parameters and longer and larger clinical trials, EPo may lead the way of adapting a safer and efficient ablation modality for the treatment of persistent AF.

## 1. Introduction

The Centers for Disease Control and Prevention in the USA reports that 1 in every 4 deaths in the United States is related to general cardiovascular disease, with an estimated 12.1 million people predicted to develop arrhythmias such as atrial fibrillation (AF) by 2030 [1]. In recent years there has been a rapid growth in the technology base and clinical appetite for targeted ablative procedures for arrhythmias, with some reports showing procedures to be effective, with quick procedural timelines, minimal associated risks and rapid recovery times [2,3]. Catheter-based ablation for AF is considered a safe and effective approach for patients who are refractory to medication. The cornerstone of catheter-based approaches to date is pulmonary vein isolation (PVI) but, increasingly, additional sites beyond the pulmonary veins are now being targeted [4]. In this review we report on the available data exploring energy-based ablative technologies, highlight the differing modalities that have been developed with a particular focus on anti-arrhythmic therapies. This review also considers the factors involved in achieving successful ablation of cardiac tissue and the evidence from in vitro and in vivo preclinical work which has informed clinical studies using EPo approaches.

## 2. Current Ablation Approaches for Treating Arrhythmia

Several relatively simple non-invasive ablative procedures have been developed to date, such as alcohol septal ablation, which involves the injection of ethanol into the septal coronary artery to target portions of the septal wall [5]. This minimally invasive ablation method has been extensively employed as a treatment for structural related heart defects such as hypertrophic cardiomyopathy, targeting the attenuation of outflow tract obstruction [2,6]. Alcohol septal ablation is often applied when previous lower intensity therapies have failed [5]. Stereotactic radioablation is another non-invasive modality under development. While not currently used in clinical practice to the best of our knowledge, a number of animal-based feasibility studies with stereotactic radioablation have been performed and reviewed elsewhere [7,8].

**Figure 1:**
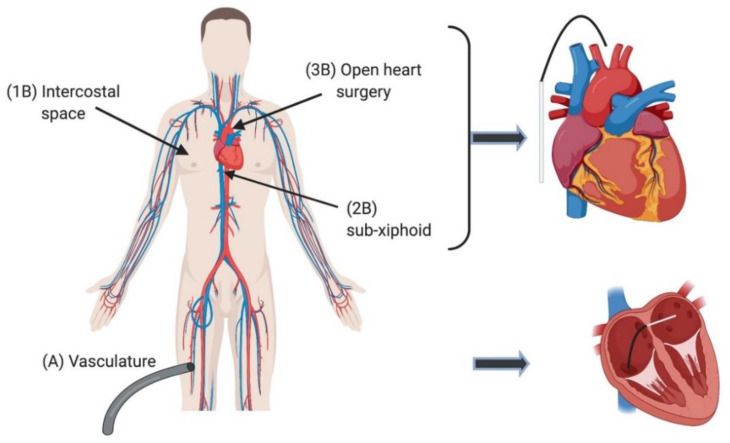
Access to the heart for invasive ablation purposes. This can be achieved via an internal endocardial approach (**A**) via the femoral vasculature (Table 1). Ablation catheter access can also be gained from an external epicardial (B) method. The extremities of the heart are reached by this technique. Access via an epicardial approach can be achieved through ports in the intercostal spaces (**1B**), a sub-xiphoid puncture (**2B**) or via open heart surgery (**3B**). The choice made between the two approaches is often made in relation to the target area and patient’s disease substrate [10].

Typically, more invasive ablation techniques require entry into the body cavity to access targeted areas of the myocardium (Figure 1). These techniques up to more recently generally involved the use of thermal energy and either induced hyper- or hypo-thermal injury at the target site [9]. Hyperthermal approaches are most commonly based on the application of radiofrequency (RF) or laser energy. Hypothermal approaches, termed cryoablation, are commonly achieved by passing cooled, thermally conductive, fluids through hollow probes at the target site.

### 2.1. Hyperthermal Techniques

Hyperthermal approaches can use various energy sources including the use of ultrasound, lasers, radiofrequency technology applied via electrode catheter, or hot balloon ablation systems [11,12]. Focused ultrasound causes the destruction of a target area due to a thermal heating effect, while remaining minimally invasive [11,13,14]. Several studies have highlighted the challenges associated with using high-intensity ultrasound for cardiac ablation [15,16,17]. Due to the incidence of oesophageal fistula and subsequent fatalities, ultrasound as a modality needs considerably more development involving lower-intensity energy and better targeting if it is to be more widely adopted [15,18]. Similar techniques are employed using lasers and have often been applied to target tumours in a variety of locations in the body [11]. Laser-based approaches employ slow heating, with low-power lasers delivered through optical fibres to induce protein denaturation. However, areas targeted by hyperthermal techniques are often difficult to control, with blood circulation proving problematic, acting to dissipate the temperature field. Some of the initial negative associations with laser or ultrasound methods are being overcome as devices become smaller and more user friendly [11]. More recent developments in commercial laser ablation systems and low-intensity ultrasound systems are beginning to compete with RF technologies in PVI applications [19].

**Figure 2:**
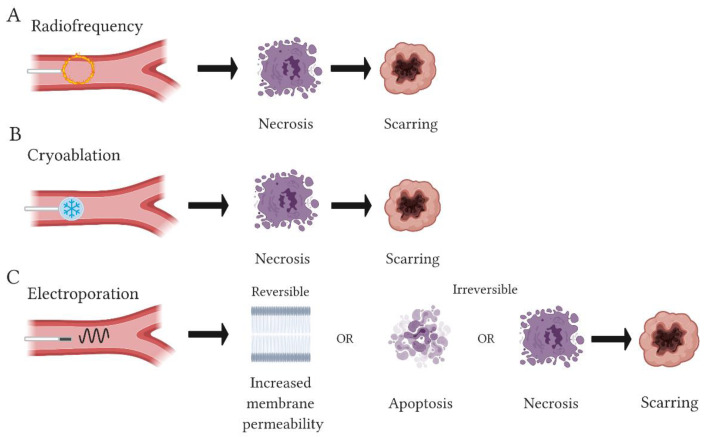
Effects of thermal and non-thermal energies. Diagram highlights the differing outcomes exhibited post-ablation between radiofrequency (**A**), cryoablation (**B**) and electroporation (EPo) (**C**) modalities. Radiofrequency and cryoablation can induce necrosis upon application which is followed by scarring with the intention to break arrhythmic circuits. Meanwhile, EPo increases membrane permeability which can lead to apoptotic or necrotic cell death, ultimately resulting in scarring.

By far the most common hyperthermal approach is based on RF technology. This modality has been in use since the 1990s and has surpassed all other energy delivery methods in popularity for use in cardiac ablation [20]. Ablation with RF relies on thermal energy from high frequency sinusoidal waves (500–750 kHz), to induce controlled damage or region-specific necrosis of heart tissue [11,20,21,22,23,24,25]. Temperatures of ≥50 °C induce tissue necrosis; however, temperatures approaching 100 °C can cause a coagulum of denatured proteins and plasma to form on the catheter tip, impeding current delivery [20] (Figure 2). RF-induced lesions typically have well-defined borders and their shape can adopt a monopolar egg shape or a bipolar round-brick shape [25,26]. RF lesion shape is dependent on a number of factors including catheter tip diameter, inter-tip spacing, tissue contact, temperature and duration of energy pulse delivered [25,26,27]. RF is a clinically significant technique as an ablation treatment for atrial fibrillation (AF), with success rates generally ranging anywhere between 45% and 80% [4,28,29].

### 2.2. Hypothermal or Cryoablation Techniques

Cryoablation has proven to be a clinically effective and a safe ablation method for use on cardiac tissue and has been studied since the early years of interventional cardiology [30]. Cryoablation uses hypothermal energy to induce ablation by freezing (≤−40 °C) [21,31] (Figure 2). Lesion shape with cryoablation is sharper, more homogeneous and less thrombogenic than lesions resulting with RF [32]. Cryoablation has been employed as a treatment particularly for arrhythmias such as AF. A study by Bárta et al. yielded success rates of approximately 90% immediately post-ablation and 48.5% of patients were free from AF at 12-month follow-up [30,33,34]. A comparison study by Kim et al. showed atrial contractility recovery rates of 32.2% and 48.8% following RF or cryoablation treatment at 12-month follow-up, respectively [35]. Similar data were reported in the FIRE and ICE clinical trial with comparable numbers of patients requiring repeat ablation procedures to sufficiently isolate pulmonary veins (PVs) and terminate arrhythmias, highlighting the success, efficacy and challenges associated with both procedures [36].

### 2.3. Challenges with Current Ablative Approaches

While there have been many positive reports particularly for RF ablation and cryoablation, their efficacies rely on the precise positioning of catheters, adequate catheter-to-tissue contact and the energy level applied to the target area [37]. Collateral damage of surrounding areas is common during the application of thermal energy, including cardiac tamponade, thromboembolism, PV stenosis, phrenic nerve and oesophageal injury or fistula, and mitral valve trauma [21,38]. Overheating of the ablation site with RF energy has resulted in ‘steam pop’ and can cause myocardial perforation or cardiac tamponade [39,40,41]. Similarly, blood flow can cause a heat sink effect during RF and cryoablation procedures, preventing uniform tissue heating or lesion formation [42]. RF has also been shown to cause coagulation within coronary vessels, induce initial hyperplasia and instigate the shrinkage of collagen fibres within the coronary arterial walls [43].

Over the last decade, the interest and demand for a more controllable and safer alternative ablation technique has been growing. The advances in electroporation (EPo) and its refinement as pulsed electric field (PEF) technology, or pulsed-field ablation (PFA), has expanded to such a degree that it can now be considered a cutting-edge, nominally thermal ablation approach. The capacity to customise parameters for further enhanced application in humans may be a turning point in the treatment of specific targeted CVDs, improving procedure management and outcomes for both clinician and patient.

## 3. Electroporation as an Ablative Approach

Catheter-based electroporation (EPo) using monophasic pulses was first employed with cardiac tissue in the 1980s but it was found to be associated with negative side effects such as the induction of an electrically isolating “vapor globe” resulting in a spark (arcing), followed by an explosion and damaging pressure waves [44,45,46]. Serious complications such as barotrauma and a pro-arrhythmic effect saw voltage-based energy systems superseded by RF ablation [46,47]. However, Ahsan et al. demonstrated that the cautious use of electroporation at lower energies could successfully avoid arcing and produce sufficient therapeutic lesions [48]. Modern voltage-based systems typically employ pulsed electric fields (PEFs) [49,50]. Ablation based on EPo is growing in popularity as an alternative to thermal ablation and causes a biophysical phenomenon to arise following the application of PEF [2,51]. These electric fields induce irreparable pore formation in cell membranes [3]. As a result, so-called PFA is considered minimally thermal and creates more predictable and controllable lesions, with minimal interaction with blood flow.

Since 2005, both irreversible (IRE) and reversible (RE) EPo has received considerable attention as a method of disrupting cell membranes for drug delivery or inducing selective cell death, respectively [11]. Both IRE and RE have the potential to be tissue-specific in terms of lethal or effective thresholds, with extracellular and endothelial structures commonly remaining intact following exposure to electric fields [52,53]. The permanent opening of nanopores in cell membranes activates intracellular molecular pathways, increases ionic and molecular transport, resulting in an overall disruption of the cell membrane and intracellular homeostasis [11,21,54]. Exposure to sufficiently large field strength results in IRE, and permanent damage and cell death ensues due to localized rearrangement within membrane structures, while supporting structures remain unscathed [9,55,56,57,58]. RE, in contrast, only transiently opens membrane pores, maintaining cell viability, and is commonly employed in the targeted delivery of drugs and nucleotides [11].

The extent and targeting of ablation with IRE can be controlled at least to some degree by changing parameters such as pulse amplitude, frequency, duration of the application and pulse number [2,59]. The lethal thresholds for many cell types have been reported based on these parameters; however, many contradictory data exist as it is still an active area of ongoing research. On the face of it, short exposures and microsecond EPo impulses can be used for biomedical applications aimed at drug delivery and gene transfer, while more prolonged impulses are related to cellular injury and ablation by IRE [55,60,61]. The shape of the applied pulse is an under-explored, and in many cases a poorly documented, parameter that has not received the same degree of experimental testing as amplitude, frequency and others (Table 1 and Table 2). Using a lung cell line, Kotnik et al. demonstrated that of the parameters used to describe pulse shape, the major factor determining electropermeabilization was the amount of time the pulse amplitude exceeded a certain threshold value [62]. They suggest that any differences observed between various pulse shapes may in fact be reflecting the difference in time the pulse is above the critical threshold for that cell type. Meanwhile, Stankevic et al. reported that it is the pulse shape and total energy input that contribute to the efficiency of IRE [63]. Sano et al. (2017) reported that asymmetric waveforms have significantly lower IRE thresholds compared to equivalent symmetrical waveforms, at least for neuroblastoma cells in vitro [64]. Both symmetrical and asymmetrical biphasic pulses have proven effective in IRE cardiac ablation procedures in both animals and a small number of pilot human trials [45,65,66,67,68]. Overall, asymmetric waveforms appear to produce more effective pore opening than symmetric pulses, possibly due to the different amplitudes of their phases. We recommend that all elements of pulse profile need to be reported, according to a set of recommended guidelines, as the extent that pulse shape contributes towards the safety and efficacy for AF treatment with IRE is unclear [69]. Overall, this is an area that requires substantial and more fundamental research before it can become part of standard clinical application [67].

More recently, the field has focused on pulse timing issues [70]. With nanosecond-PEFs in particular, this has been shown to improve the controllability of pore size. Short duration nsPEFs have been shown to minimise the electrophoretic effects associated with cell membrane transport [70,71]. When compared with longer pulse durations, shorter durations are reported to limit solute movement, overall reducing the osmotic imbalance and improving cell targeting with PEF exposure. nsPEF stimuli are too short to induce capacitive charging and instead aim to influence displacement currents over conduction currents [70]. Elementally, every cell behaves independently, deeming intercellular electric connections ineffective on membrane charging [72]. However, the mechanism by which such short stimuli can influence pore opening is still not fully understood and is the subject of ongoing research [70].

### 3.1. Pre-Clinical Evaluation of IRE, towards Optimization of Parameters for Clinical Use

Ex vivo studies were a milestone in the adaptation of IRE for in vivo applications as early work by Krassowska et al. emphasized the formation of pores in tissue exposed to EPo in a 2D model of cardiac tissue [73]. Selective pore formation can prevent excessively high transmembrane potentials, limiting damage in surrounding tissues. As work progressed, studies investigated the therapeutic thresholds and biophysical effects of EPo at a cellular level [3]. This was achieved by altering some therapeutic variables (pulse duration, pulse frequency, amplitude) and comparing the induction of injury on tissue through lactate dehydrogenase activity and the integrity of cell membranes.

Experiments involving the murine atrial cardiac cell line HL-1, cultured as adherent monolayers, showed that the damage was proportional to the number of IRE pulses and field strength applied [3] (Table 1). When compared to previous work from the same group looking at the human prostate cancer line LNCaP, data suggested that cardiac cells were more suspectable to IRE at higher field strengths greater than 1000 V/cm. Kaminska et al. showed that pulse intensities above 375 V/cm were destructive in the immature rat H9C2 myoblast cell line [74] (Table 1). A scan of the potential range of field strengths that might induce cell death is required and would be enhanced by the addition of threshold data on neuronal, cardiomyocyte and fat tissue found in the heart. Very recently, Hunter et al. showed that cardiac cells are more susceptible to electroporation damage than cortical neurons and oesophageal smooth muscle cells [75]. However, there are very few reports of this nature examining the different IRE thresholds of cardiac cells relative to other appropriate cardiac–neuronal model systems.

In animal studies, the application of IRE to cardiac tissue for the treatment of arrhythmias was found sufficient to block aberrant conductive pathways and reduce conduction and propagation of disruptive electrical signals [76]. In a study by Zager et al., it was shown that longer pulse durations (100 µs versus 70 µs) and increased pulse number (20 versus 10) were associated with a larger volume of damage in the ventricular myocardium in a rat model [2]. In a porcine model, the controlled delivery of electrical pulses both monophasic and biphasic, over a few microseconds or nanoseconds, has been shown to create tissue injury while avoiding negative effects [45].

**Figure 3:**
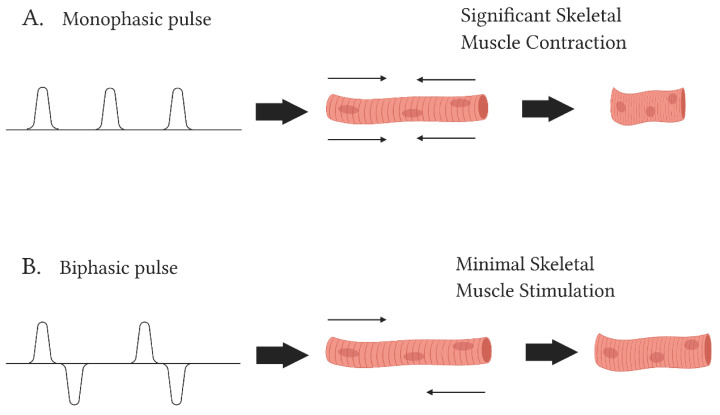
Structure of monophasic and biphasic pulses and their effect on muscle during ablation. (**A**) Monophasic pulses have been shown to induce excessive skeletal muscle spasm in patients thus requiring the use of general anaesthesia and paralytics. (**B**) Muscle activation induced by biphasic is minimal and therefore requires sedation.

In terms of pulse polarity, studies have found that biphasic pulses show better efficacy than monophasic stimuli in penetrating epicardial fat and overcomes the impedance by fatty cells during ablation of cardiac tissue [43,60]. Similarly, while both monophasic and biphasic pulses have proven efficient at producing feasible ablation outcomes, biphasic waveforms have been shown to create more durable lesions than monophasic applications [77]. This may be owing to biphasic pulses significantly altering electric field bias, reducing ion charging and prolonged post-ablation depolarizations [70]. Due to a “cancellation effect”, higher amplitudes are often required to achieve ablation when using biphasic shocks at the nanosecond level [60]. Ablation success is seen to be influenced by the time between successive pulses. Nanosecond pulses can induce a uniform activation of the myocardium by forming a consistent electric field distribution [70]. This has been found to reduce the risk of new wave-fronts arising that could reinitiate arrhythmia and fibrillation [70]. Studies have also demonstrated that patients receiving monophasic pulses commonly require general anaesthesia and neuromuscular paralytics during procedures [78]. In comparison, patients that received biphasic pulses or high frequency energy were able to have the procedure under conscious sedation due to minimal resulting skeletal muscle activation [60,67,78] (Figure 3). It has been proposed that direct current (DC) monophasic energy be replaced by short alternating current (AC) biphasic energy to target larger areas, as it appears to reduce capture of nearby excitable tissues, thus reducing muscle spasms and acute pain during ablation [79].

### 3.2. Controlled Lesion Formation with IRE In Vivo

Human studies using comparably greater pulse durations and frequencies, ranging from microseconds to milliseconds (Table 1), show that the ablative effect and lesion area depends on the electric resistivity of the tissue, presence of cell membranes and the applied electric field [47]. Short electric field pulses cause rapid lesion formation, which is favorable to procedural work-flow [68]. However, the rapid nature of the delivery of IRE provides little, if any, opportunity for clinicians to change position of catheters or the profile of the energy delivered during the active phase of energy delivery.

While clinical outcome reports for IRE are limited, they are growing, and success has been noted in early clinical trials. Reddy et al. and Loh et al. have in a series of papers shown the safety and efficacy of IRE in the clinic [68,80,81]. Firstly, the authors highlighted the safety of the IRE procedure by successful acute pulmonary vein isolation (PVI) in 100% of patients [68,78,81]. This was a turning point in IRE ablation as it highlighted the potential of IRE and its capability to replace current thermal techniques in PVI procedures. Freedom from AF was later recorded in 94.4 ± 3.2% of patients by Reddy et al., in a recent trial using a either a combined RF/IRE (pulsed-field) approach or IRE as a standalone ablation procedure [80]. Similarly, 100% of PVs in patients with symptomatic paroxysmal or persistent AF were successfully isolated by Loh et al., using IRE alone [81].

Compared to thermal approaches, IRE appears to be less reliant on specific anatomical catheter positioning or catheter–tissue contact to produce adequate lesions, however this has not been examined systemically and requires more evidential data [51]. Successful ablations have been demonstrated even when delivering less precise, more widespread energy, suggesting that tissue vulnerability and lesion formation depends on tissue susceptibility and tissue type, facilitating cell-specific targeting with controlled parameter selection [51]. Studies have shown that IRE lesions occur locally in regions directly associated with electrodes [82,83]. Regions surrounding the electrodes are exposed to lower electric fields which induce RE, thus cells in this region recover and revert to normal function. Whether IRE lesions are transmural or not varies with the increasing thickness of the myocardium, requiring lesions to be wider to ensure adequate penetration [84]. IRE-induced lesions of the myocardium can be observed at a cellular level and are differentiated from unaffected tissue by a sharp border, similar to those induced by RF and cryoablation [9]. Appropriate transmural lesions are necessary to ensure the isolation of targeted regions to prevent disease relapse, thus avoiding the need for repeated procedures [9].

It has been suggested that IRE parameters can be fine-tuned to achieve different lesion profiles [78]. The data to date, suggest that lesion geometry is significantly influenced by a number of pulse parameters and electrode spacing, with lesion size and depth corresponding mainly to the magnitude of field strength delivered [40,72,85] (Table 1). Early studies in which IRE was applied to porcine tissue noted that no charring or tissue disruption was visible upon gross inspection, and a clear demarcation line was evident around electroporated regions at the cellular level [9]. Further histological inspection demonstrated that while avoiding a significant local temperature change, successful electrical isolation was evident in the atria and was accompanied by transmural destruction. In addition, these studies demonstrated that lower field strengths can create sufficient lesions on PVs, while higher energies result in tissue shrinkage of the ventricle [53,86]. In porcine models, IRE-induced lesions were found to be similar to those formed with RF energy, however microscopic analysis revealed that IRE lesions have reduced epicardial fat-associated inflammation and fewer intralesional sequestered cardiomyocytes [45]. Furthermore, fibrotic regions formed during remodeling at the site of energy delivery were more homogenous in those areas exposed to IRE than to RF energy [45]. As expected, both modalities were linked to neointimal thickening on the undisrupted endocardium, fibrosis of intralesional nerves, and absence of endocardial thrombus formation. Similar histological results were recorded in canine studies upon the targeting of epicardial ganglia with IRE, highlighting preservation of cardiomyocyte architecture, minimal inflammatory response and fibrosis [76]. Studies have also shown that blood has a higher conductivity than tissues, which may affect IRE lesion depth of procedures done using an endocardial approach; however, unlike thermal techniques, this interaction with blood does not cause coagulation [87]. While substantial information has been collected from animal models, evidence of lesion geometry in humans cannot be assessed to such degrees. Instead, lesions are observed from a gross, clinical perspective and their electrical conduction is monitored during procedures. The inspection of the effect of IRE on vasculature is commonly achieved via imaging techniques.

### 3.3. Advantages and Disadvantages of EP

Preclinical and clinical data overall support the efficacy and safety of IRE with its capacity to limit injury to surrounding structures [51,68,73,74]. Due to the importance of protecting the coronary vasculature, several studies have investigated the short-term (3 weeks) and longer-term (3 months) effects of IRE on blood vessels (Table 1). Du Pre et al. investigated the effect of IRE when applied directly to the coronary vasculature in a porcine model. They observed that the coronary vessels remained free from clinically relevant damage, regardless of the lesion proximity to the vessel [43]. This further supports the targeting benefits of IRE which is not influenced by arterial blood flow, even when applied directly to vessels [43]. Long-term follow-up studies of porcine models by Neven et al. also highlighted the safety of IRE when targeted at the coronary vasculature [44]. They demonstrated that even deep lesions had no effect on luminal coronary artery diameter in their long-term study, proving IRE to be a safe ablation strategy for use on or in close approximation to the coronary vasculature.

The minimal damage IRE induces to vasculature makes it an attractive ablation modality for further development [77]. The major drawback with current thermal techniques is the induction of vessel stenosis, particularly of the PVs. The mechanism behind PV stenosis is believed to be a combination of intimal hyperplasia, medial thickening and smooth muscle activation, which is often followed by scar retraction and vein narrowing [88]. Comparative studies have drawn interesting comparisons between the use of RF energy and IRE for ablation of PVs. Early investigations were conducted on porcine models showing the effect of the given energies on inducing PV stenosis [89] (Table 1). A study revealed the significant damage caused by RF on PV tissue with observations of necrotic myocardium, large amounts of scar tissue surrounding the myocardial sleeve and intimal and elastic lamina proliferation. In contrast, only minor intimal proliferation was noted on tissue targeted with IRE. An initial decrease in PV diameter was noted in IRE experiments, however later studies revealed an overall increase in diameter [89]. It was evident that no PV stenosis arose due to IRE-exposed subjects at 3-month follow-up, while those who underwent RF ablation exhibited stenosis immediately that persisted for the follow-up survival period [89]. In recent years, the effects of IRE and RF were investigated in humans and research showed that the incidence of stenosis and narrowing of PV diameter following PFA was virtually eliminated (0%), while patients who received RF energy saw a 12.0% reduction in diameter and 32.5% incidence of stenosis at 3-month follow-up [90].

Some early preclinical work on porcine PVs by Wittkampf et al. reported that lower field strengths can create feasible and safe lesions on PV ostia, without evidence of collateral damage to surrounding structures [53,91] (Table 1). Preservation of nerve tissue was observed with no significant damage to the phrenic nerve post-IRE procedure [92]. With the oesophagus lying near the heart, the avoidance of trauma here is also a major concern of electrophysiologists. Similar findings were reported in a canine model, with subjects showing no signs of oesophageal injury [93]. In a porcine model, Neven et al. reported no disruption to oesophagus architecture, even with purposeful targeting of the adventitia [94]. This highlights the feasibility of IRE applications for cardiac tissue. In the first human trials IMPULSE and PEFCAT, incorporating the FARAPULSE endocardial ablation system, combined analysis by Reddy et al. showed the tissue-specific nature of IRE [78]. There were no reports of oesophageal or phrenic nerve injury in patients receiving the therapy, demonstrating that IRE possesses a major safety advantage over thermal techniques as an ablation modality for cardiac tissue [78].

Another major difference between IRE and thermal catheter-based procedures is the time taken to perform the procedure. From a practical perspective, IRE procedures require significantly less time, energy and number of applications of energy in comparison to those of RF or cryoablation [3,59,72,78,93]. In the FIRE and ICE trial, studies by Reddy et al., on the isolation of PVs, highlighted a notable difference in mean total procedure time for IRE (92.2 min) compared to RF and cryoablation procedure times, which required 141 and 124 min, respectively [78]. Similarly, left atrial catheter-dwell time was much lower in IRE procedures (34 min) in contrast to RF and cryoablation (109 and 92 min, respectively) [78]. Similar results have been recorded in AF treatments with cryoablation yielding significantly shorter procedure times (ranging between 73.5 ± 16 min to 192.9 ± 44 min), in comparison to RF energy techniques (from 118.5 ± 15 min to 283.7 ± 78.0 min), with IRE procedures yielding even less time overall (from 22.6 ± 8.3 min to 92.2 ± 27.4 min) [78,80,95]. Shorter procedure times also incorporate less fluoroscopy duration, reducing a patient’s radiation exposure [96]. While procedure time is not a crucial factor in ablation, shorter duration can enhance productivity by reducing healthcare costs overall. Therefore, IRE has a clear advantage over current procedures for efficacy, safety and reduced procedure times.

While IRE overcomes many complications associated with thermal ablation techniques, it does pose some similar risks such as thrombosis, haemorrhage and infection, however these are common to all procedures employing similar access techniques [50]. Specific to IRE there is an associated risk of electrolysis when untuned current is passed through body fluids with dissolved electrolytes, instigating gas formation [97,98]. One study reported that different current polarity may decrease gas bubble formation as a side-effect of IRE, highlighting that a reduced number of gas bubbles are released when using anodal IRE, compared to RF or cathodal IRE [98]. Gaseous microemboli could result in myocardial damage and in some instances with symptomatic cerebral ischemic events due to the obstruction of capillaries [98]. However, the risk of microemboli appears very low with preclinical canine reports by Neven et al. detailing that no treatment-related cerebral events occurred due to gas formation during IRE procedures [99]. In addition, MRI imaging and histopathology confirmed the absence of cerebral emboli, supporting the safety of this procedure [99].

The most immediate effects of electric field application to the myocardium are electrophysiological, leading to possible changes in ECG, such as in the ST segment and T-wave, or an overall decrease in resting cell membrane potential [50]. In some instances, the use of PEFs in non-cardiac applications has also been involved in the evolution of lethal and non-lethal cardiac arrhythmias in animal studies [51]. To minimise the risk of induced arrhythmia, it is presumed that ECG synchronisation integrated with pulse delivery is critical to ensure the energy is applied only during the absolute refractory period of the heart to avoid a critical increase in cell permeability [66,82,100]. A recent clinical trial by Loh et al. incorporated ECG synchronisation for the delivery of monophasic IRE pulses [81]. During this study, no peri-procedural complications were recorded. Likewise, in a study by Reddy et al. no adverse effects were reported when using biphasic pulses without synchronization to depolarization of the atria or ventricles [80]. Thus, the absolute requirement for synchronization is unclear. The use of IRE requires meticulous monitoring of blood pressure and electrolytes, as instances of induced mild hypertension and epileptic-like seizures have been reported with the use of high voltages and general anaesthesia during IRE procedures [81,90,101]. Cases of such intraoperative complications could jeopardize patient safety, therefore a clear understanding and rapid, appropriate management by the clinician is paramount. Nevertheless, it has been concluded that an application of irreversible PEFs directly to cardiac tissue both endocardially and epicardially in preclinical studies is safe when timed with the R-wave during sinus rhythm, and in early clinical studies regardless of timing [51,66,80,81,82,90,100,101]. However, as PEF is a novel technique, safety boundaries and significant safety data remain scarce for human application and further investigation is required.

## 4. Conclusions

IRE has seen its stock rise substantially as a therapeutic intervention in recent decades and there has been much interest in its safety and feasibility for use on cardiac tissue. While significant advances have been made based on animal studies, particularly involving porcine and canine models, and preliminary parameters have been developed for use in humans (Table 2), much optimisation remains to be achieved. Further testing and fine-tuning are required to adapt and potentially individualise these parameters for specific patients or patient groups, while ensuring precise delivery of energy to achieve efficient EP ablation. There is significant room for the development of more complex representative in vitro model systems that incorporate both functional and histological outcomes, that are multi-cellular and more easily translatable. This will facilitate rapid development of pulse parameters and potentially catheter design by looking at the catheter not just to deliver energy, but to also provide feedback on target site and success of the ablation.

Similarly, while there are substantial preclinical data for IRE from animal models, the number of clinical trials is limited. Studies completed to date include small cohorts of approximately eighty patients with varying follow-up times of 3, 4 and 12 months [78,80,90]. Therefore, not only larger, multicentre trials are required to analyse the effects of IRE but also long-term evaluation of the permanence of the ablation.

Lesions are difficult to investigate in human studies, thus, most information is to be acquired regarding the true depth and volume of lesions is collected from animal studies. Follow-up times of preclinical trials generally exceed no longer than 3 or 4 months (Table 1). Similarly, long-term studies would challenge the durability of lesions in humans and examine any relapse to the electrical or structural induced CVD originally treated by IRE. Another limitation to current IRE trials is the lack of consistency between experiments. Some studies are limited to one energy magnitude, while others either use smaller or greater magnitudes on different sized animals (Table 1). While there are few published clinical trials related to the use of IRE on cardiac tissue, preclinical studies provide a promising baseline representation of its use. IRE bypasses many of the complications and drawbacks of the more commonly used thermal ablation modalities. With further improvements and refinement of parameter specifics, IRE may prove to be the gold standard for ablative CVD therapy.

## Figures and Tables

**Table 1:** Comparison of preclinical IRE studies on cardiac tissue.

Ref.	Subject	Follow-Up	Energy	Parameters	Monophasic/Biphasic Waveform	Monopolar/Bipolar Electrode Configuration	Reported Outcome
**In Vitro**							
[3]	HL-1 cell line	N/A	200 V; 1000 V/cm	PD- 50 µs, F- 10 Hz, PF- 10, 50, 99 pulses.	Not specified	Not specified	(1) IRE is effective for creating lesions on HL-1 cell line.
[73]	Cardiac strand-2D model	N/A	0.4–0.5 V; 25 V/cm	PD- 5 ms	Monophasic	Not specified	(1) Cardiac fibre exposed to a strong stimulus responds by developing pores in the first layer of cells immediately adjacent to the electrode. (2) IRE stops the growth of the macroscopic transmembrane potential, it does not affect intra- and extracellular potentials in the bulk of the tissue.
**In Vivo Animal**							
[2]	Rat	1 month	50, 250, 500 V	PD- 70 vs. 100 μs, F- 1, 2, 3, 4 Hz, PF- 10 V’s 20.	Not specified	Not specified	(1) Longer pulse duration (100 μs vs. 70 μs) is associated with larger volume reduction. (2) More pulses (20 vs. 10) are associated with larger volume reduction. (3) Pulse voltage (500 V vs. 250 V, 50 V) has an important effect on tissue damage. (4) Lower pulse frequency (10 Hz vs 20 Hz) is correlated with harsher tissue damage.
[9]	Porcine	24 h	1500–2000 V	PD- 100 μs, PF- 8, 16, 32.	Not specified	Not specified	(1) Lesions were mean 0.9 cm in depth. (2) Complete transmural destruction of atrial tissue at the site of the electrode application. (3) No local temperature change and with demonstration of electrical isolation.
[40]	Porcine	7 days	Not specified	F- 1 Hz, PF- 35	Not specified	Bipolar	(1) Unlike RF lesions, SW lesions showed only mild denaturation and little disruption of endocardium. (2) Lesion depth from SW correlated to amount of energy used. (3) SWCA lesions showed transient inflammatory responses followed by accelerated healing process with preserved myocardial blood flow.
[43]	Porcine	3 weeks	Not specified	Not specified	Monophasic	Not specified	(1) Mean depths ranged from 2.9 + 1.2 mm–6.5 + 2.7 mm. (2) 32% of lesions were transmural. (3) Coronary arteries do not develop significant stenosis within 3 weeks after epicardial IRE.
[44]	Porcine	3 months	Not specified	PF- 3.	Monophasic	Not specified	(1) Mean value of the median lesion depths was 6.4 ± 2.6 mm. (2) 31% of lesions were transmural. (3) Apart from short-lasting (<30 min) coronary spasm, no long-term luminal narrowing was seen.
[45]	Porcine	2 weeks	500 V	PD- 90 µs, PF- 60.	Biphasic	Bipolar	(1) PFA lesions comparable to RFA lesions and had no collateral damage.
[51]	Canine	29 days	750 V	PD- 20 µs, F- 30–500 Hz, PF-10.	Not specified	Bipolar	(1) PEF can safely ablate Purkinje fibres. (2) Minimal collateral damage to myocardium.
[53]	Porcine	3 weeks	Not specified	PF- 4.	Monophasic	Bipolar	(1) Low energy IRE is safe and efficient in creating lesions on the PV ostia.
[57]	Rat	N/A	20 kV; 36 kV/cm	PD- 10 ns, F- 2 Hz, PF- 3.	Not specified	Not specified	(1) nsEP produces smaller pore size and reduced non-polar distribution of electro-pores over the cell body. (2) At near threshold intensities, both nsEPo and msEPo triggered Ca^2+^ transients.
[58]	Rabbit	N/A	50–500 V	F- 1–2 kHz, PF- 6–10.	Monophasic	Bipolar	(1) IRE thresholds were 229 ± 81 and 318 ± 84 V for the endocardium and the epicardium, respectively. (2) Selective transient impairment of electrical activity in endocardial bundles is caused by IRE. (3) IRE might transiently reduce myocardial vulnerability to arrhythmias.
[59]	Ovine	N/A	Not specified	PD- 100–400 µs, F- 1–5 Hz, PF- 10–40 pulses.	Not specified	Bipolar	(1) Lesions were well demarcated from the unaffected tissue. (2) The induced inflammatory reaction within these acute ablations was minimal.
[67]	Porcine	3 weeks	600 V	PD- 2 ms, F- 10 kHz, PF- 10.	Biphasic	Not specified	(1) Demonstrated the feasibility of a novel asymmetrical high frequency (aHF) waveform for IRE. (2) The aHF waveform led to significantly deeper lesions than the symmetrical HF waveform. (3) Both methods showed lesions of more than 4 mm deep.
[70]	Murine, rat, porcine	N/A	100 V; 12.2 kV/cm	PD- 400 ns, PF- 20.	Not specified	Not specified	(1) Stimulation by 200 ns shocks can elicit Ca^2+^ transients. (2) Shortest shocks cause the least damage and their threshold energy is minimal. (3) Orientation of cardiomyocytes with respect for electric field does not affect threshold for ns shocks.
[71]	Murine	N/A	Not specified	PD- 200 µs	Not specified	Not specified	(1) 200 ns stimuli induced action potentials. (2) nsPEF caused Ca^2+^ entry, associated with a slow sustained depolarisation.
[72]	Rabbit	N/A	200 V	PD- 350 ns, F- 1, 3 Hz, PF- 20, 6.	Not specified	Monopolar	(1) Nonconducting lesions created in less than 2 s with nsPEF application per site and minimal heating (<0.2 °C) of the tissue. (2) Lesion was smoother and more uniform throughout the wall in comparison to RF lesions.
[76]	Canine	113 ± 7 days	1000 V	PD- 100 µs, PF- 10	Not specified	Bipolar	(1) Cardiac GP permanently damaged using DC for IRE. (2) Preservation of atrial myocardial architecture and absence of inflammatory reaction and fibrosis.
[77]	Porcine	63 ± 3.3 days	800–1800 V	Not specified	Monophasic	Bipolar	(1) Both waveforms created confluent myocardial lesions. (2) Biphasic PFA was more durable than monophasic PFA and radiofrequency ablation lesions.
[83]	Rabbit	4 weeks	300 V	Not specified	Monophasic	Bipolar	(1) Shock-induced IRE was spatially dependent on the location and dimension of the active region of the shock electrode. (2) The surviving anterior epicardial layers in the infarcted region were more susceptible to IRE.
[84]	Rabbit	Not specified	200 V; 3 kV/cm	PD- 350 ns, F- 3 Hz, PF- 6.	Not specified	Not specified	(1) High anisotropy ratio substantially affects the ablation outcome, low anisotropy ratio does not.
[85]	Porcine	3 months	Not specified	Not specified	Monophasic	Not specified	(1) Lesion size, depth and width corresponds to magnitude of energy used. (2) Initial spasm of coronary vasculature was noted, but this did not persist and was not recorded at follow-up.
[86]	Porcine	3 months	Not specified	Not specified	Not specified	Not specified	(1) Mean depth of the 30 J, 100 J and 300 J lesions was 3.2 ± 0.7, 6.3 ± 1.8 and 8.0 ± 1.5 mm, respectively. (2) Mean width of the 30 J, 100 J, and 300 J lesions was 10.1 ± 0.8, 15.1 ± 1.5 and 17.1 ± 1.3 mm, respectively. (3) No luminal arterial narrowing was observed after 3 months.
[87]	Porcine	3 weeks	950–2150 V	PD- <10 ms, PF- 4.	Monophasic	Monopolar	(1) 200 J applications yielded median lesion depth of 5.2 ± 1.2 mm. (2) No signs of tissue heating. (3) Lesion would be sufficient for inducing PVI.
[88]	Canine	N/A	Not specified	PD- 60–300 s, F- 7 kHz.	Not specified	Not specified	(1) Device can successfully deliver both RF and IRE energy. (2) Addition of porous configuration on balloon can aid in enhancing drug delivery.
[89]	Porcine	3 months	Not specified	Not specified	Monophasic	Not specified	(1) IRE ablation: PV ostial diameter decreased 11 ± 10% directly after ablation but had increased 19 ± 11% after 3 months. (2) RF ablation: PV ostial diameter decreased 23 ± 15% directly after ablation and remained 7 ± 17% smaller after 3 months than pre-ablation diameter, despite a 21 ± 7% increase in heart size during aging from 6 to 9 months.
[91]	Canine	N/A	Not specified	F- 1 Hz.	Not specified	Bipolar	(1) No evidence of collateral damage to surrounding structures. (2) Ventricular arrhythmias can occur during DC application and are more likely with use of higher energy.
[93]	Canine	27 days	2 kV/cm	PD- 100 µs, PF- 100.	Not specified	Bipolar	(1) No significant PV stenosis or oesophageal injury occurred.
[3]	Porcine	N/A	500 V; 1200 V/cm	PD- 50 µs, F- 10 Hz, PF- 50.	Not specified	Not specified	(1) IREis effective for creating lesions on PV tissue.
[102]	Porcine	35 days	2200 V	PD- <60 s	Biphasic	Bipolar	(1) Fibrous tissue homogeneously replaced myocytes. (2) When present, nerve fascicles and vasculature were preserved within surrounding fibrosis.
[103]	Canine ex vivo	N/A	750–2500 V; 250–833 V/cm	PD- 200 µs, F- 1 Hz, PF- 10	Biphasic	Not specified	(1) Delivery of IRE energy significantly reduced the window of vulnerability to ventricular arrhythmia. (2) No evidence of myocardial damage.

**Table 2:** Comparison of clinical IRE studies on cardiac tissue.

Ref.	Follow-Up	Energy	Parameters	Monophasic/Biphasic Waveform	Monopolar/Bipolar Electrode Configuration	Reported Outcome Reported Outcome
[68]	N/A	900–2500 V	PF- 3.	Not specified	Bipolar	(1) PEF is a safe method for treating AF both endocardially and epicardially. (2) No incidences of atrial or ventricular arrythmia during procedure. (3) No collateral damage or PV stenosis recorded.
[78]	4 months	900–1000 V	Not specified	Monophasic	Bipolar	(1) Acute PVI achieved in 100% of patients using 6.4 ± 2.3 applications. (2) No injury to oesophagus or phrenic nerve.
[80]	12 months	0.011 ± 0.006 mV	PD- 3–5 s	Biphasic	Bipolar	(1) No adverse effects recorded related to PEF. (2) Freedom from AF was 94.4 ± 3.2%.
[81]	N/A	2154 ± 59 V	Not specified	Monophasic	Monopolar	(1) Acute bidirectional electrical PVI achieved in all 40 PVs. (2) No PV reconnections occurred during waiting period (30 min).
[90]	3 months	900–1000 V	Not specified	Monophasic	Monopolar and Bipolar	(1) No change (0%) in PV diameter and no stenosis in PFA patients, but reduction in diameter in 32.5% of patients who received RFA.

## Data Availability

Not applicable.
